# Supplementary material for: A Scoping Review of Acute Sedentary Behaviour Studies of People with Spinal Cord Injury
Source: Int J Environ Res Public Health. 2024 Oct 18;21(10):1380. doi: 10.3390/ijerph21101380 (PMC11507420; doi:10.3390/ijerph21101380)
Supplement: Supplementary file 1 [file ijerph-21-01380-s001.zip › SB & SCI_Supplement1.pdf]

Search Databases

Medline, CINAHL, Embase, and PsycInfo databases will be searched using relevant keywords. The reference lists of identified studies and relevant review articles will also be examined. Studies not written in the English language will be excluded.

Search Terms

	Term	Entered Search Terms
**Population**	Individuals with Spinal Cord Injury	“spinal cord*” OR “spinal adj4 injur*” OR “spin* lesion*” OR “spinal impairment*” OR “spine adj3 impairment” OR “spine injur*” OR “conus medullaris syndrome” OR “spine transection*” OR “spine impairment*” OR “brown Sequard*” OR “cauda equina syndrome*” OR “posterior cord syndrome*” OR “myelitis” or “myoplagia” OR “autonomic dysreflexia” OR paralysis” or “parapleg*” OR “tetrapleg*” or "motor complete*" or "motor incomplete*"<br>exp Spinal Cord Injuries
**Concept 1**<br><br>OR<br><br>**Concept 2**	Psychological<br><br>OR<br><br>Physiological	“attitude*” OR “perception*” OR “emotion*” OR “motivation*” OR “biopsychosocial*” OR “enjoy*” OR “arousal” OR “pleasure” OR “displeasure” OR “cognition” OR “executive function” OR “cognit*” OR “executive function*” OR “feeling*” OR “self efficacy” OR “affect” OR “affective symptoms”<br>exp Emotions<br>exp stress, psychological<br>exp Health Behavior<br>exp Illness Behavior<br><br>“cardiovascular*” OR “metabolic*” OR “cardiometabolic*” OR “vascular*” OR “heart*” OR “muscular*” OR “neuromuscular*” OR “endocrine*” OR “blood pressure*” OR “systolic*” OR “diastolic*” OR “arter*” OR “arterial stiffness*” OR “lipids*” OR “cholesterol*” OR “triglycerides*” OR “glucose*” OR “insulin*” OR “endothelial adj4 function*” OR “cardiac diseases”<br>exp heart disease<br>exp Cardiovascular Diseases<br>exp Metabolic Diseases<br>exp Nutrition Disorders
**Context**	Sedentary Behavior	“sedentary behavi*” OR “sedent*” OR “sitting*” OR “lying*” OR “reclin*” OR “supine*” OR “sitting adj3 interrupt*” OR “light physical activity*” OR *“24-hour movement behavior*” OR “24 hour movement behavior”* OR “ecological momentary analysis”

Types of Study to be Included

Randomized controlled studies, randomized crossover trials, quasi-experimental, pilot studies, thesis/dissertation studies, case-control studies, qualitative studies, surveys, observational studies.

Condition or domain to be studied

Acute sedentary behavior (> 1 hr)

Participants/Population

Inclusion: Adults with spinal cord injuries. Studies will be included if sedentary behavior is measured, analyzed, quantified, discussed or used as a stimuli.

Exclusion: People with disabilities that are not spinal cord injuries. Studies that evaluate long term physical inactivity or a “sedentary lifestyle.” Studies that attempt to promote physical activity and merely mention sedentary behavior as a state to be avoided or contraindicated by the intervention within a study.

Intervention(s)/Exposure(s)

Studies that quantify sedentary behavior using accelerometry, self-reported surveys, ecological momentary analysis, or calculation of 24 hour movement behaviors.

Psychological analysis of the perceptions, attitudes, and conceptions or engaging in acute sedentary behaviors such as sitting, lying, or reclining while exerting < 1.5 METs.

Physiological measurement of cardiovascular, metabolic, muscular, neurological, or endocrine measurements during an acute bout (>1 hr) of sedentary behavior.

Comparator(s)/control

For a scoping review, there will not be a comparator. We will be reviewing any studies and work to determine the state of literature regarding the research question and topic.

Main outcome(s)

The purpose of this project is to review all outcomes related to SB, not to determine a main outcome. The goal is to address these topics in a broad and “scoping” format.
