# Supplementary material for: A Scoping Review of Acute Sedentary Behaviour Studies of People with Spinal Cord Injury
Source: Int J Environ Res Public Health. 2024 Oct 18;21(10):1380. doi: 10.3390/ijerph21101380 (PMC11507420; doi:10.3390/ijerph21101380)
Supplement: Supplementary file 1 [file ijerph-21-01380-s001.zip › SB & SCI_Supplement2.pdf]

STUDY ID		Study	Bailey	Nooijen	Postma	Sonenblum	Kawasaki	Kooner	Wecht	Wecht
		Study ID	1	2	3	4	5	6	7	8
		Year	2020	2015	2020	2016	2012	1988	2006	2009
DEMOGRAPHIC INFORMATION	n	sample (n)	14	37	47	28	11	12	18	5
		female (n)	8	5	22	6	0	0	0	0
		female (n, %)	57.14	13.51	46.81	21.43	0.00	0.00	0.00	0.00
	AGE	age (mean)	51		54.5	41	32.7		39	40
		age (median)		44						
		age (SD)			12.9	12			*7.5*	10
		age (95 CI)	9							
		age (SEM)					2.1			
		age (IQR low)		30						
		age (IQR high)		56						
	BODY MASS / BMI	BMI							*25.9*	25.6
		BMI (SD)							*6.4*	6.7
		Body Mass (kg)	87.9				61.3		*83*	78
		Body Mass (kg, SD)							*25*	23
		Body Mass (kg, CI)	22.7							
		Body Mass (kg, SEM)					3.2			
		Body Mass (lb)				175				
		Body Mass (lb, SD)				45				
DESCRIPTION		General Study Category	Physiological, Psychological	Psychological, Behavioural	Behavioural	Behavioural	Physiological	Physiological	Physiological	Physiological
		Comparison	Uninterrupted sitting v. Interrupted sitting	Relations of SB with ESE	SB changes over time after Rehabilitation	Sitting Behaviour, Including PR and WS	2 h of sitting after 2 h of exercise	People w/ Tetraplegia, Sitting v. Recumbency	Fit v. Unift People with SCI Recovering from Exercise	LNAME v. Placebo Injection, supine (HUT, less sig.)

**PHYSIOLOGICAL OUTCOMES**	Postprandial Glucose iAUC	Signficant							
	Postprandial Glucose tAUC	Significant							
	Postprandial Insulin iAUC	NS							
	Postprandial Insulin tAUC	NS							
	Postprandial Triglyceride iAUC	NS							
	Postprandial Triglyceride tAUC	NS							
	Blood Glucose iAUC	NS							
	Blood Glucose tAUC	NS							
	Plasma insulin iAUC	NS							
	Plasma Insulin tAUC	NS							
	Triglyceride iAUC	NS							
	Triglyceride tAUC	NS							
	Mean Arterial Pressure	NS							Significant
	Systolic Blood Pressure	NS				NS	Significant		Significant
	Diastolic Blood Pressure	NS				NS	Significant		Significant
	Plasma Albumin Post-Exercise					Significant			
	Total Protein Post-Exercise					Significant			
	Fluid Changes after in response to exercise and resting					NS*			
	Plasma Sodium					NS			
	Plasma osmolality					Significant			
	Urinary Volume					NS	NS		
	Urinary Sodium Excretion						NS		
	Urinary potassium excretion						Significant		
	Plasma Renin Activity						NS		Significant
	Plasma Aldosterone						NS		Significant
	Heart Rate						Significant		

	Vagal Recovery from Exercise							Significant Group Main Effect	Significant
	Combined Autonomic Control							Significant	
	Sympathovagal Balance							Significant	
	HR Recovery from Exercise							NS	
PSYCHOLOGICAL	National Well-being Measurement	NS							
	Warwick Edinburgh Mental Well-Being Scale	NS							
	Short Positive and Negative Affect Scale	Significant							
	Schwarzer and Renner Physical Exercise Self-Efficacy Scale	NS	NS						
	Fatigue (Visual Analogue Scale)	NS							
	SB x ESE		NS						
	Motility x ESE		NS						
	PA x ESE		Significant						
BEHAVIOURAL	Sedentary Day-Time hr/ day		1.55						
	Motility (g)		16.2						
	Wheeled PA hr / day		1.12						
	Sedentary Time @ Discharge hr / day			12.20666667					
	Sedentary Time 6 months after Discharge hr / day			11.04666667					
	Sedentary Time 12 months after Discharge hr / day			11.08333333					

	Prolonged SB >30 min hours / day @ discharge			7.59					
	Prolonged SB >30 min hours / day 6 mos after discharge			7.105					
	Prolonged SB >30 min hours / day 12 mos discharge			7.003333333					
	% of people who transfer to a recliner/couch				75%				
	Avg. Time in chair (h)				10.6				
	Mean transfers out of chair				8.4				
	Mean PR / hr of occupancy				0.4				
	Mean WS frequency /hr of occupancy				2.4				
	Average upright sitting segment without PR or WS (mins)				140.0				
	Ambulation Level x SB			Significant					
	Age at Discharge x. SB			Approaching Significance					
	Time since Injury x SB			NS					
	Interaction time since injury x time (discharge to 6 mos) x SB			Significant					
	Interaction lesion level x time since discharge x SB			NS					
